# Patient-Centered Digital Screener for Elder Abuse in the Emergency Department

**DOI:** 10.1093/geroni/igab046.580

**Published:** 2021-12-17

**Authors:** Fuad Abujarad, Esther Choo, James Dziura, Chelsea Edwards, Michael Pantalon, Karen Jubanyik, Gail D'Onofrio, Thomas Gill

**Affiliations:** 1 Yale University, New Haven, Connecticut, United States; 2 Oregon Health & Science University, Portland, Oregon, United States; 3 Yale University, New H, Connecticut, United States; 4 Yale University School of Medicine, New Haven, Connecticut, United States; 5 Yale University School of Medicine, Yale University School of Medicine, Connecticut, United States

## Abstract

Elder abuse is a growing problem where many cases are left unidentified by professionals. For some older adults, the emergency department may be the sole point of care where they have an opportunity to be identified as victims of abuse. However, current methods of screening tend to miss less obvious forms of abuse and may deter older adults from self-reporting due to either a lack of understanding of abuse or fear of potential consequences. VOICES is an innovative, self-administrated, and automated tablet-based tool that combines screening, educational content, and brief motivational interviewing to enhance and improve identification of elder abuse cases. Combining an elder abuse screener and digital coach designed to guide the older adult through a customized pathway to encourage self-identification and self-reporting of abuse, VOICES is a robust tool engineered to place the screening process in the hands of the older adults, rather than the providers. We will discuss preliminary results of the ongoing feasibility study currently being conducted in the ED, which has successfully enrolled over 500 older adults. Current data indicate that 93% of patients find the tool to be satisfying, engaging, and easy to use. Preliminary findings also suggest that older adults who come in with “Little to none” knowledge of elder abuse increase knowledge of abuse after using the tool. In summary, VOICES appears to be a feasible tablet-based screening tool in the emergency department.

